# Associations Between Left Ventricular Dysfunction and Brain Structure and Function: Findings From the SABRE (Southall and Brent Revisited) Study

**DOI:** 10.1161/JAHA.116.004898

**Published:** 2017-04-18

**Authors:** Chloe M. Park, Emily D. Williams, Nish Chaturvedi, Therese Tillin, Robert J. Stewart, Marcus Richards, Dean Shibata, Jamil Mayet, Alun D. Hughes

**Affiliations:** ^1^ UCL Institute of Cardiovascular Science University College London London United Kingdom; ^2^ Institute of Psychiatry, Psychology and Neuroscience King's College London London United Kingdom; ^3^ MRC Unit for Lifelong Health and Ageing at UCL London United Kingdom; ^4^ Department of Radiology University of Washington Seattle WA; ^5^ ICCH Imperial College London London United Kingdom

**Keywords:** arterial stiffness, brain imaging, brain volumes, cognitive function, cognitive impairment, left ventricular dysfunction, left ventricular dysfunction, microvascular dysfunction, Clinical Studies, Echocardiography, Cognitive Impairment

## Abstract

**Background:**

Subclinical left ventricular (LV) dysfunction has been inconsistently associated with early cognitive impairment, and mechanistic pathways have been poorly considered. We investigated the cross‐sectional relationship between LV dysfunction and structural/functional measures of the brain and explored the role of potential mechanisms.

**Method and Results:**

A total of 1338 individuals (69±6 years) from the Southall and Brent Revisited study underwent echocardiography for systolic (tissue Doppler imaging peak systolic wave) and diastolic (left atrial diameter) assessment. Cognitive function was assessed and total and hippocampal brain volumes were measured by magnetic resonance imaging. Global LV function was assessed by circulating N‐terminal pro–brain natriuretic peptide. The role of potential mechanistic pathways of arterial stiffness, atherosclerosis, microvascular disease, and inflammation were explored. After adjusting for age, sex, and ethnicity, lower systolic function was associated with lower total brain (beta±standard error, 14.9±3.2 cm^3^; *P*<0.0001) and hippocampal volumes (0.05±0.02 cm^3^, *P*=0.01). Reduced diastolic function was associated with poorer working memory (−0.21±0.07, *P*=0.004) and fluency scores (−0.18±0.08, *P*=0.02). Reduced global LV function was associated with smaller hippocampal volume (−0.10±0.03 cm^3^, *P*=0.004) and adverse visual memory (−0.076±0.03, *P*=0.02) and processing speed (0.063±0.02, *P*=0.006) scores. Separate adjustment for concomitant cardiovascular risk factors attenuated associations with hippocampal volume and fluency only. Further adjustment for the alternative pathways of microvascular disease or arterial stiffness attenuated the relationship between global LV function and visual memory.

**Conclusions:**

In a community‐based sample of older people, measures of LV function were associated with structural/functional measures of the brain. These associations were not wholly explained by concomitant risk factors or potential mechanistic pathways.

## Introduction

There is an established link between heart failure (HF) and impaired cognitive function, with the prevalence of mild cognitive impairment in HF patients as high as 53%.^1^ Subclinical left ventricular (LV) dysfunction has also been associated with early cognitive impairment; however, findings are inconsistent. While some studies have shown associations between impaired cognition and LV systolic dysfunction,^2^, ^3^ one investigation found stronger relationships with LV diastolic dysfunction.^4^

Volumetric brain analyses by cerebral magnetic resonance imaging (MRI) have shown a close association between reduced hippocampal volume and cognitive impairment.^5^, ^6^ Yet, results from studies associating cardiac function with cerebral MRI findings are conflicting, with grey matter loss in the hippocampal regions related to LV dysfunction in some studies^7^ and not others.^8^, ^9^

Inconsistencies in associations between LV dysfunction and cognitive status may be explained by selection of study populations, with some samples chosen according to disease or risk status,^4^ as well as range of measurement of LV and brain indices.^2^, ^4^, ^8^

Importantly, previous studies have failed to test whether the LV function‐cognition relationship is the result of confounding by other pathophysiological mechanisms, such as arterial stiffness, microvascular damage, atherosclerosis, and inflammation. These mechanisms are known to be adversely associated both with LV function^10^, ^11^ and cognition^12^, ^13^; thus, observed associations between LV function and cognition may be confounded by “common causes.” Understanding these pathways is essential to identifying cardiac targets that may delay or prevent cognitive decline and dementia.

We aimed to combine comprehensive and sensitive measures of global, systolic, and diastolic LV function, with MRI and cognition data in a community‐based, multiethnic cross‐sectional sample of older people, as well as investigate explanatory mechanisms. We hypothesized that adverse LV function would be associated with low brain volumes and cognitive dysfunction. We additionally adjusted for cardiometabolic risk factors and separately examined the role of: (1) arterial stiffness, (2) atherosclerosis, (3) microvascular disease, and (4) inflammation in accounting for any observed associations between LV function and cognitive measures.

## Methods

### Study Population

The SABRE (Southall And Brent REvisited) study is a tri‐ethnic population‐based cohort consisting of white European (2346), first‐generation migrant South Asian (1711), and African Caribbean (801) men and women. Cohort details have been previously described^14^ In brief, men and women aged 40 to 69 years from the West London areas of Southall and Brent were recruited from general practitioners' lists and from workers in local industries. Sampling was stratified by ethnicity, sex, and age group. Exclusions included conditions that would preclude informed consent or attendance at a local clinic. The target sample was sent letter invitations from their general practitioner. Positive respondents were contacted by phone to discuss the study and sent participation information sheets before attending local clinics for investigation.

Surviving participants were invited to attend the 20‐year follow‐up investigation between 2008 and 2011. The study was conducted at the Imperial Biomedical Research Centre, London. The study was approved by St Mary's Hospital Research Ethics Committee (07/H0712/109) and all participants provided written informed consent. The study adheres to the principles of the Declaration of Helsinki and Title 45, US Code of Federal Regulations, Part 46, Protection of Human Subjects, Revised November 13, 2001, effective December 13, 2001.

### Investigations

At the follow‐up clinic, participants fasted and refrained from alcohol, smoking, and caffeine for at least 12 hours before attendance, omitting medication on the clinic morning. Participants completed a questionnaire detailing sociodemographic characteristics (including years of education as a measure of socioeconomic position), health behaviors, medical history, and medication. Height, weight, and waist circumference were measured as previously described.^14^ Waist‐hip ratio was calculated. Seated brachial blood pressure was measured after 10 minutes of rest using an Omron 705IT (Omron Healthcare, Kyoto, Japan). An appropriately sized cuff was placed on the left upper arm, 3 recordings were taken 2 minutes apart, and the second and third recordings were averaged. Hypertension was defined as use of blood pressure–lowering medication from patient questionnaire and general practitioners' medical record review. We also included individuals who had a clinic systolic blood pressure >140 mm Hg or diastolic blood pressure >90 mm Hg. Established diabetes mellitus was defined by medical records. Participants without known diabetes mellitus underwent an oral glucose tolerance test, and unrecognized diabetes mellitus was defined according to World Health Organization 1999 guidelines.^15^ Coronary artery disease was defined as a coronary event or revascularization identified by hospital admissions and medical record review and adjudicated by an independent committee. Stroke diagnoses was based on predetermined criteria of symptoms, duration of symptoms, and MRI or computed tomography imaging from hospital admission, patient, or medical records.^16^ HF and atrial fibrillation were identified during clinic visit and/or via medical records.

#### Cognitive function

Cognitive assessments were conducted by trained staff on the morning of investigation to avoid fatigue. The battery comprised tests previously validated for cross‐cultural settings.^17^, ^18^ The following domains were derived by summing standardized individual test scores: global/overall function (Community Screening Instrument for Dementia [CSID] cognitive assessment)^19^; verbal memory (immediate and delayed verbal recall [CERAD 10‐word]^20^), visual memory (picture recognition); fluency (animal naming^21^), processing speed (color trail‐making A and B^22^); and working memory (forward and backward digit span^23^).

#### Magnetic Resonance Imaging

##### Total and hippocampal brain volume

Cerebral MRI was performed based on the Cardiovascular Health Study protocol.^24^ This included sagittal T1‐weighted and axial T1‐weighted, proton density, and T2‐weighted images of 5‐mm thickness with no gaps. For volumetric measures, 3‐mm axial fluid‐attenuated inversion recovery and coronal 1.5‐mm 3‐dimensional T1‐weighted gradient echo images were also obtained. A third of the scans were performed on a General Electric Signa HDxt 1.5T scanner and the rest on a General Electric Discovery MR750 3T scanner (GE Healthcare, Waukesha, WI)

An automated segmentation protocol was used to quantify total brain and hippocampal volume using FIRST in FSL 4.1.^25^ Total brain volume (TBV) was computed as the volume after skull stripping of the T1‐weighted image using BET^26^ in FSL 5.0. Hippocampal volumes were calculated as the sum of right and left segmented volumes. Brain volumes and cognitive function domains were standardized into *z* scores.

##### White matter hyperintensities

Assessment of white matter hyperintensities was performed by one grader (methods have been previously described^27^). Severity of white matter hyperintensities was scored on a 10‐point scale combining periventricular and subcortical foci. White matter grades were grouped into the following categories: none for grade 0, mild for grade 1, moderate for grade 2, and severe for grades at least 3.

##### Echocardiography

Echocardiography was performed using a Philips iE33 ultrasound machine (Philips, Amsterdam, The Netherlands) with a 5.0 to 1.0 phased array transducer (S5‐1), as previously described.^28^, ^29^ LV mass was calculated according to American Society of Echocardiography guidelines and indexed to height^2.7^. Left atrial diameter was measured using 2‐dimensional echocardiography and indexed to height. LV hypertrophy was classified according to American Society of Echocardiography guidelines. Transmitral flow velocity during the early filling phase (E) and the late filling phase (A) were acquired by pulsed Doppler and averaged from 3 consecutive cycles. Tissue Doppler imaging was used in pulsed wave mode to measure longitudinal tissue velocities. The sample volume was placed at the mitral annulus of the lateral and septal LV wall. Peak velocities during systole (Sa), early diastole (Ea), and late diastole (Aa) were averaged from 3 consecutive cycles and averaged. The ratio of the transmitral early and late filling phases (E:A) and the ratio of early and late tissue Doppler imaging peak velocities (Ea/Aa) were calculated as measures of diastolic function. The ratio of early filling and early myocardial velocity (E/Ea) was calculated as a noninvasive index of LV filling pressure. Diastolic dysfunction was classified according to American Society of Echocardiography guidelines.

Stroke Volume (SV)=End‐diastolic volume (EDV)−End‐systolic volume (ESV)

Ejection Fraction (EF)=SV/EDV

Cardiac Output (CO)=SV×Heart Rate

Cardiac Index (CI)=CO/BSA

##### Retinal photography

Retinal photography was performed using a Zeiss FF450+ fundus camera (Oberkochen, Germany), and 30° digital images of the disc (ETDRS Field 1), macula (ETDRS Field 2), temporal to macular region (ETDRS Field 3), and superior‐temporal region (ETDRS Field 4) were captured using an Allied Vision Tech Oscar 510C CCD (2588×1958 pixels; Singapore) following pupillary dilation with tropicamide (1%) and phenylephrine (2.5%). Digital retinal photography was performed on 1205 participants, and analyzable images were obtained in 1185 individuals. Images were graded for retinopathy and classified as none, mild, moderate, or severe/proliferative according to the NHS Diabetic Eye Screening Programme feature‐based grading classification http://diabeticeye.screening.nhs.uk/gradingcriteria.

##### Blood and urine analysis

Serum N‐terminal pro–brain natriuretic peptide (NT‐proBNP) was measured using an Elecsys 2010 electrochemiluminescence analyzer (Roche Diagnostics, Burgess Hill, UK) calibrated using the manufacturer's reagents. A combination of serum creatinine and cystatin C concentrations were used to calculate estimated glomerular filtration rate, measured using an automated analyzer (c311; Roche Diagnostics, Burgess Hill, UK). Serum interleukin‐6 was measured using a multiplex electrochemiluminescence immunoassay (MSD, Rockville, MD). A spot early‐morning urine sample was measured for albumin and creatinine and the albumin:creatinine ratio was calculated.

### Statistical Analysis

Statistical analyses were performed using Stata version 13.0 (StatCorp, College Station, TX). Participant characteristics for continuous data are reported as mean±SD, or median (interquartile range) for skewed data, and number (percentage) for categorical data. Full echocardiography data are presented in Table 1; however, 3 key LV measures were selected a priori for analysis. For systolic function, the key measure was the tissue Doppler imaging systolic wave (Sa), which was chosen for its precision and sensitivity to early long‐axis systolic dysfunction compared with ejection fraction.^30^ For diastolic function, left atrial diameter indexed to height was chosen because it provides a long‐term indication of the effects of chronically elevated filling pressure. NT‐proBNP levels represented global dysfunction.^31^ With the exception of diastolic dysfunction grade, all LV measures are presented as continuous variables. Multivariable regression analyses assessed the associations of markers of LV systolic, diastolic, and global function with cognitive function scores and brain volume (total and hippocampal). Sex and ethnicity interactions were tested and were nonsignificant, therefore all data were pooled, with sex and ethnicity adjustment in all models. Model 1 adjusted for age, sex, and ethnicity. Model 2 additionally adjusted for concomitant cardiometabolic risk factors, diabetes mellitus, hypertension, previous stroke, coronary artery disease, waist‐hip ratio, years of education, and smoking.

**Table 1:** Characteristics of All Participants Who Underwent Echocardiography

No.	1338
Men, No. (%)	1011 (76)
Ethnicity, No. (%)	
White European	632 (47)
South Asian	488 (37)
African Caribbean	218 (16)
Age, y	69.6±6.1
Body mass index, kg/m^2^	27.4±4.5
Waist‐hip ratio	
Men	1.00±0.06
Women	0.92±0.08
Hypertension, No. (%)	890 (67)
Diabetes mellitus, No. (%)	409 (31)
Coronary artery disease, No. (%)	324 (24)
Stroke, No. (%)	77 (6)
Heart failure, No. (%)	38 (3)
Atrial fibrillation, No. (%)	92 (7)
Smoking, No. (%)	
Never	757 (57)
Ever	491 (37)
Current	83 (6)
Systolic blood pressure, mm Hg	140±18
Diastolic blood pressure, mm Hg	77±10
Mean arterial pressure, mm Hg	98±11
Education, y	11.8±3.3
MRI brain volumes, cm^3^	
Hippocampal volume	7.4±1.0
Total brain volume	1426±174
LV global function	
NT‐proBNP, pg/mL	85 (47, 170)
LV systolic function	
Ejection fraction, %	61.6±9.5
Ejection fraction <55%, No. (%)	274 (21)
Cardiac index, L/min per m^2^	2.06±0.5
Sa, cm/s	7.5±1.4
LV diastolic function, cm/s	
Mitral E	64±17
E/Ea	9.4±3.1
E:A	0.86±0.25
E/Aa	6.4±2.2
Ea/Aa	0.7±0.2
Normal diastolic function, No. (%)	77 (6)
Mild diastolic dysfunction, No. (%)	948 (76)
Moderate diastolic dysfunction, No. (%)	220 (18)
Severe diastolic dysfunction, No. (%)	1 (0)
LADI, cm/m	0.024±0.003
LV structure	
LVMI, g/m^2.7^	44.0±11.9
LVH, No. (%)	304 (23)
EDV, mL	93.7±23.2
Vascular and other measures	
cfPWV, m/s	11.4±3.5
cIMT, mm	0.82±0.2
Microvascular disease	
ACR	3.03±16.8
eGFR, mg/mL	73.4±16.2
Retinopathy, No. (%)	
None	741 (67)
Mild	335 (30)
Moderate/proliferative	35 (3)
WMH	
Grade 0	23 (2)
Grade 1	438 (35)
Grade 2	381 (30)
Grade 3	416 (33)
IL‐6, pg/mL	1.70±2.7

Data are expressed as mean±SD or median (25th–75th percentile for skewed data) for numerical data and number (percentage) for categorical data. A indicates mitral inflow late wave; Aa, tissue Doppler imaging peak late diastolic wave; ACR, albumin:creatinine ratio; cfPWV, carotid to femoral pulse wave velocity; cIMT, carotid intima‐media thickness; Ea, tissue Doppler imaging peak early diastolic wave; EDV, end‐diastolic volume; eGFR, estimated glomerular filtration rate; IL‐6, interleukin 6; LADI, left atrial diameter indexed to height; LV, left ventricular; LVH, left ventricular hypertrophy; LVMI, left ventricular mass indexed to height^2.7^; Mitral E, mitral inflow early wave; MRI, magnetic resonance imaging; NT‐proBNP, N‐terminal pro–brain natriuretic peptide; Sa, tissue Doppler imaging peak systolic wave; WMH, white matter hyperintensities.

#### Potential mechanisms

The following mechanisms were separately added to model 2 to understand their possible role in associations between key LV measures and cognitive function/brain volumes: (1) arterial stiffness was assessed by carotid to femoral pulse wave velocity using the Micro‐Medical Pulse Trace system (PT2000; Rochester, Kent, UK); (2) far wall left common carotid intima‐media thickness imaged using a Philips iE33 ultrasound machine with a 11‐3 MHz linear transducer was used as a measure of subclinical atherosclerosis; (3) a global measure of microvascular disease was calculated as a composite *z* score of estimated glomerular filtration rate, albumin:creatinine ratio, white matter hyperintensities, and retinal photography grade (these were chosen because they correlate with MRI‐measured cerebral microvascular disease^32^); and (4) interleukin‐6 was chosen as our principal inflammatory biomarker; however, additional sensitivity analyses were conducted using interleukin‐1β, interferon‐γ, tumor necrosis factor‐α, C‐reactive protein, and glycoprotein acetyls as alternative inflammatory markers. These alternative biomarkers in models did not substantially alter findings and the respective models are therefore not shown. Significance was assigned at *P*<0.05.

## Results

**Figure 1:**
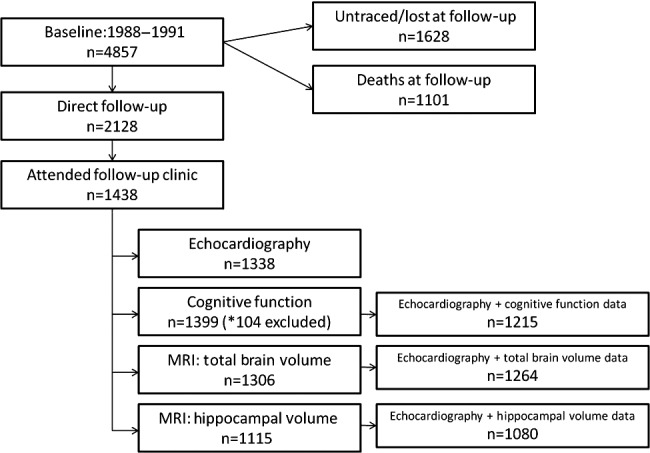
Flow chart showing sample sizes for our key measures. *A total of 104 cognitive function tests were excluded for reasons that may have affected cognitive assessment, eg, impaired hearing/eyesight. MRI indicates magnetic resonance imaging.

Of the 1438 participants who returned for follow‐up investigations, a total of 1338 had full echocardiography. Of those who had echocardiography, 1215 individuals also had full cognitive data, 1264 had TBV measures, and 1080 had hippocampal brain volume measures. Figure 1 is a flow diagram showing sample sizes for our key measures.

### Participant Characteristics

Participant characteristics are presented in Table 1. The mean (SD) age of the sample was 69±6 years. A total of 76% of participants were men, 47% were white European, 37% were South Asian, and 16% were African Caribbean. Hypertension was highly prevalent (67%), while 31% of patients had diabetes mellitus, 24% had coronary artery disease, 6% experienced a stroke, 7% had atrial fibrillation, and 3% had HF.

### Associations Between Left Ventricular Function and Brain Volumes

Associations between LV function and brain volumes are presented in Table 2. After adjusting for age, sex, and ethnicity (model 1), poorer LV systolic function was significantly associated with smaller TBV and hippocampal volume. A 1‐cm/s decrease in Sa was associated with a 1% smaller TBV (14.9±3.2 cm^3^, *P*<0.0001) and a 0.68% smaller hippocampal volume (0.05±0.02 cm^3^, *P*=0.01). The association with TBV remained unchanged after adjustment for cardiometabolic disease, education, and smoking (model 2: 14.9±3.3 cm^3^, *P*<0.0001), while the association with hippocampal volume was slightly attenuated (model 2: 0.04±0.02 cm^3^, *P*=0.07). A significant association was also observed between greater NT‐proBNP and smaller hippocampal volume. Per‐unit increase of NT‐proBNP was associated with a 1.35% smaller hippocampal volume (−0.10±0.03 cm^3^, *P*=0.004). This association was reduced after multivariate adjustment (−0.07±0.04 cm^3^, *P*=0.06). Associations between other LV measures and brain volumes are presented in Table S1.

**Table 2:** Associations Between LV Function and Brain Volumes and Cognitive Function

	Sa, cm/s				LADI, cm/m				NT‐proBNP, pg/mL			
	Model 1		Model 2		Model 1		Model 2		Model 1		Model 2	
	β±SE	*P* Value	β±SE	*P* Value	β±SE	*P* Value	β±SE	*P* Value	β±SE	*P* Value	β±SE	*P* Value
TBV, cm^3^	14.9±3.2	<0.0001	14.9±3.3	<0.0001	−3.25±14	0.8	−0.51±15	0.97	1.18±5.2	0.8	1.61±5.6	0.8
Hippocampal volume, cm^3^	0.05±0.02	0.01	0.04±0.02	0.07	−0.04±0.09	0.7	0.03±0.1	0.7	−0.10±0.03	0.004	−0.07±0.04	0.06
CSID (*z* score)	0.003±0.01	0.8	0.009±0.01	0.6	−0.023±0.07	0.7	−0.01±0.07	0.9	−0.007±0.02	0.8	−0.004±0.02	0.9
Verbal memory (*z* score)	0.023±0.01	0.1	0.022±0.01	0.1	−0.09±0.07	0.2	−0.03±0.07	0.7	−0.045±0.03	0.07	−0.020±0.03	0.4
Visual memory (*z* score)	−0.0052±0.02	0.8	−0.0047±0.02	0.8	0.09±0.09	0.3	0.13±0.10	0.2	−0.076±0.03	0.02	−0.083±0.04	0.02
Working memory (*z* score)	−0.003±0.02	0.9	0.0009±.02	0.96	−0.21±0.07	0.004	−0.19±0.08	0.01	−0.033±0.03	0.2	−0.026±0.03	0.4
Fluency (*z* score)	0.031±0.02	0.08	0.030±0.02	0.07	−0.18±0.08	0.02	−0.14±0.08	0.08	−0.043±0.03	0.1	−0.036±0.03	0.2
Processing speed (*z* score)	0.003±0.01	0.8	0.0015±0.01	0.9	0.08±0.06	0.2	0.03±0.06	0.7	0.063±0.02	0.006	0.049±0.02	0.04

Data are expressed as β±standard error (SE) (cm^3^) for brain volumes and β±SE (*z* scores) for cognitive domains. Model 1 was adjusted for age, sex, and ethnicity. Model 2 was additionally adjusted for diabetes mellitus, hypertension, previous stroke, coronary artery disease, waist to hip ratio, years of education, and smoking. CSID indicates Community Screening Instrument for Dementia; LADI, left atrial diameter indexed to height; LV, left ventricular; NT‐proBNP, N‐terminal pro–brain natriuretic peptide; Sa, tissue Doppler peak velocity in early systole; TBV, total brain volume.

### Associations Between Left Ventricular and Cognitive Function

In Table 2, model 1, Sa was not significantly associated with any measure of cognitive function. An increase in left atrial diameter indexed to height was adversely associated with working memory (−0.21±0.07, *P*=0.004) and fluency (−0.18±0.08, *P*=0.02). The association with working memory persisted after further adjustment for confounders (Table 2, model 2; −0.19±0.08 [*P*=0.01]). Increased levels of NT‐proBNP were adversely associated with visual memory (−0.076±0.03, *P*=0.02) and processing speed scores (0.063±0.02, *P*=0.006). Further adjustment for confounders (model 2) did not significantly alter these associations.

Associations between other LV measures and cognitive function are presented in Table S1.

### Sensitivity Analyses

Sensitivity analyses were performed to test the associations between brain volumes and LV function adjusting for height. In a separate analysis, patients with prior stroke, atrial fibrillation, and HF were excluded (n=177); all results were unaltered (results not shown).

### Potential Mechanisms

**Figure 2:**
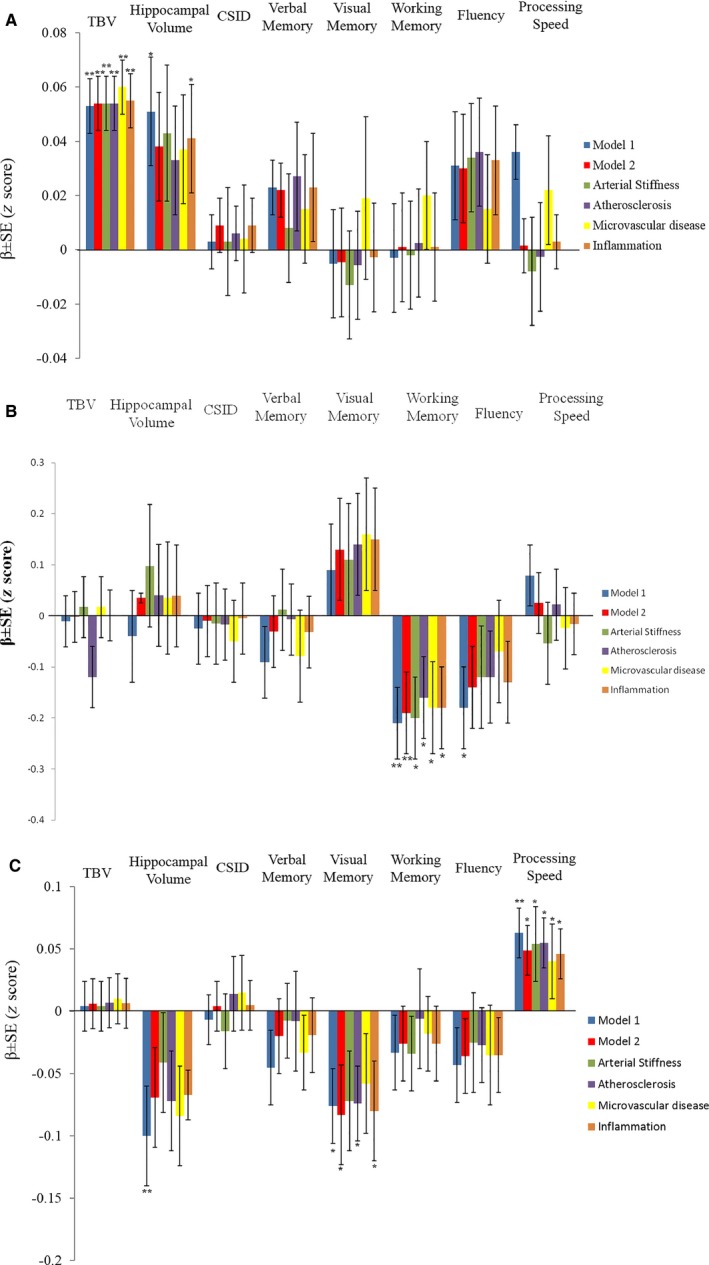
Bar charts showing the effect sizes in the relationships between total brain volume (TBV), hippocampal volume, Community Screening Instrument for Dementia (CSID), verbal memory, visual memory, working memory, fluency, and processing speed with (A) systolic (Sa), (B) diastolic (LADI), and (C) global (N‐terminal pro–brain natriuretic peptide) function. To facilitate comparison across brain volumes and cognitive tests, all of the results on the y‐axis are displayed as mean β±standard error (SE) change in standardized values (*z* scores). Model 1 was adjusted for age, sex, and ethnicity. Model 2 was additionally adjusted for diabetes mellitus, hypertension, previous stroke, coronary artery disease, waist to hip ratio, years of education, and smoking. Possible mechanisms are investigated by adjusting for all of the above‐mentioned risk factors (model 2) plus further separate adjustment for: (1) arterial stiffness, (2) atherosclerosis, (3) microvascular disease, and (4) inflammation. *P<0.05, **P<0.01.

Arterial stiffness, atherosclerosis, microvascular disease, and inflammation were separately added to model 2. Separate adjustment for any of these measures did not alter the significant association between Sa and TBV (Figure 2A), left atrial diameter indexed to height, and working memory (Figure 2B), or NT‐proBNP and processing speed (Figure 2C). However, the association between NT‐proBNP and visual memory was attenuated by adjustment for either arterial stiffness or microvascular disease (Figure 2C).

## Discussion

In a community‐based, multiethnic sample, we found robust relationships between global, systolic and diastolic LV dysfunction, and functional and structural measures of the brain. Microvascular disease and greater arterial stiffness appeared to partly explain some of the relationships; however, the residual link between LV dysfunction and brain measures could suggest a direct influence of LV dysfunction on brain structure and function.

Normal aging is often associated with cognitive decline and reductions in brain volume;^33^ these processes are accelerated in the presence of cardiovascular disease and its risk factors, although distinguishing between age‐related and disease‐related cognitive decline is very difficult.^34^ The study objective was to investigate brain structure and function in a community sample, and although the majority of participants would not have fulfilled criteria for dementia, the findings are important for our understanding of the full spectrum of brain impairment.

### Relationship Between Left Ventricular Function and Brain Volumes

We show a robust association between LV systolic function and TBV, unexplained by concomitant risk factors or known mechanistic pathways. A 1‐cm/s decrease in Sa was associated with a 1% smaller TBV (14.9±3.2 cm^3^, *P*<0.0001), which corresponds to the equivalent volume change observed with normal aging over 3.1 years.^35^ This relationship is consistent with existing literature.^8^, ^36^ We expand on previous results by showing an association with Sa, a more sensitive measure of systolic function,^37^ and by investigating the role of alternative pathways. Furthermore, while previous studies investigated associations with LV systolic function, we additionally found no association between TBV and LV diastolic function or levels of NT‐proBNP.

Although losses in regional brain volume associated with dementia are poorly understood, hippocampal atrophy has been found to be an important predictor of progression from mild cognitive impairment to Alzheimer's disease^38^ and is closely linked with amnesic subtypes of mild cognitive impairment.^27^ We found associations between measures of systolic and global LV function and hippocampal volume, consistent with findings of hippocampal atrophy among patients with HF.^7^ A 1‐unit decrease in systolic function was associated with a 0.68% smaller hippocampal volume (0.05±0.02 cm^3^, *P*=0.01), and a 1‐unit decline in global function was associated with a 1.35% smaller hippocampal volume (−0.10±0.03 cm^3^, *P*=0.004), which corresponds to the equivalent volume change observed with normal aging over 0.8 and 1.6 years, respectively.^35^ These associations were reduced, however, by adjustment for concomitant risk factors. Our lack of other volumetric brain measures prevents us from contributing to the discussion of other regions' relative importance in the relationship between cardiac and cognitive function.

### Relationship Between Left Ventricular Function and Domains of Cognitive Function

We assessed global/overall cognitive function, as well as the domains of verbal memory, visual memory, working memory, fluency, and processing speed, since various cognitive modalities have previously been differentially associated with LV function.^2^, ^4^, ^8^ While we show no significant associations between Sa and cognitive function, we observed strong associations between LV diastolic function and working memory and fluency, and LV global function and visual memory and processing speed. With the exception of diastolic function and fluency, these associations persisted after adjustment for cardiovascular risk factors. The level of decline observed in working memory and fluency per 1‐unit decline in diastolic function corresponds to the equivalent decline observed with normal aging over 4.3 and 21.2 years. The level of decline observed in visual memory and processing speed per 1‐unit decline in global function corresponds to the equivalent decline observed with normal aging over 1.6 and 1.4 years, respectively.^39^ These results are in agreement with the Hoorn study, which previously highlighted the important role of global and diastolic LV function on cognitive function.^4^ In contrast to our results, the previous study reported stronger associations with the domains of attention and executive functioning, rather than memory. To the best of our knowledge, we are the first to report an association between diastolic function and working memory. The lack of an association in the Hoorn study might be due to the grouping of several different subdomains of memory.

### Potential Mechanisms

Despite calls for more work,^40^, ^41^ limited research has investigated the pathways explaining the LV dysfunction‐cognition relationship. In this study, we examined alternative mechanisms that might explain our observed relationships.^42^ We conservatively conceptualized them as confounding; however, it is possible that they may also act on the causal pathway between LV function and brain structure and function. The attenuation of the relationship between global LV function and visual memory following arterial stiffness or microvascular disease adjustment indicates confounding, likely due to the common influence of these confounders on both LV and cognitive dysfunction.

The residual link between LV dysfunction and brain measures after adjustment for these pathways could suggest a direct influence of LV dysfunction on both brain structure and function, most likely to involve impaired cerebral perfusion under stressor conditions. Hypoperfusion, through various mechanisms, has been proposed as a probable candidate for structural and functional brain changes,^34^, ^43^, ^44^ most likely through reduced cardiac output for systolic dysfunction. Further research is required to clarify the causative role of diastolic dysfunction; however, it has recently been associated with the development and progression of white matter lesions, relating to heightened risks of cognitive decline.

### Study Strengths

This study builds on previous literature by examining a range of sensitive measures of global, systolic, and diastolic LV function, using rigorous laboratory controls and protocols. In particular, Sa is a highly sensitive and accurate measure of systolic LV function, superior to ejection fraction/stroke index, which only capture disturbed function at a later stage.^30^ We have expanded previous work by exploring the mechanisms through which LV function might affect brain structure/function and alternative competing pathways. Our results from this community‐based, multiethnic study of subclinical LV dysfunction provide generalizability beyond previous work in this area,^2^, ^4^, ^8^ which had studied predominantly white, high‐risk populations. The prevalence of HF was low in our sample (3%) and therefore the results are unlikely to be driven by clinical‐level dysfunction.

### Study Limitations

Despite the study strengths, there are some limitations to consider. Our cross‐sectional data preclude conclusions regarding the direction of underlying causal relationships and we cannot rule out the possibility of reverse causality. Confounding factors at one time point does not take into account cumulative or past exposure and we cannot account for confounding due to factors that have not been measured. We also acknowledge that multiple comparisons were made, increasing the possibility of a false‐positive finding. Furthermore, loss to follow‐up means that the group may be subject to attrition and survival bias; however, by definition, assessment of later‐life cardiovascular and cognitive function necessitates survival into old age. As is common in health research,^45^ healthier and socioeconomically advantaged participants were more likely to attend, leading to a possible underestimation of the magnitude of the associations observed, given that the least healthy participants were less likely to have chosen/been able to participate or to have survived to follow‐up.

## Conclusions

We have shown an association between LV dysfunction and functional and structural measures of the brain that is not fully explained by concomitant risk factors or alternative mechanistic pathways. Prospective longitudinal studies are required to fully understand the nature of the relationship between LV function and abnormal brain structure and function. The clinical implications of reduced cognition among people with impaired LV function are considerable. Many cognitive processes including executive function, memory, and reasoning are needed to successfully manage chronic disease medication regimens and to maintain independence in older age.^34^ Identification of cognitive risk factors that can be targeted for early modification are of considerable clinical significance for delaying and even preventing dementia in our aging population.

## Sources of Funding

This work was supported by the Wellcome Trust (082464/Z/07/Z) and the British Heart Foundation (SP/07/001/23603, PG/08/103, and PG/12/29/29497).

## Disclosures

None.

## Supporting information


**Table S1.** Associations Between All Left Ventricular Function Variables and Brain Volumes and Cognitive Function
