# Supplementary material for: Associations Between Left Ventricular Dysfunction and Brain Structure and Function: Findings From the SABRE (Southall and Brent Revisited) Study
Source: J Am Heart Assoc. 2017 Apr 18;6(4):e004898. doi: 10.1161/JAHA.116.004898 (PMC5533007; doi:10.1161/JAHA.116.004898)
Supplement: Supplementary file 1 — Table S1. Associations Between All Left Ventricular Function Variables and Brain Volumes and Cognitive Function [file JAH3-6-e004898-s001.pdf]

Supplemental Material

Table S1. Associations between all left ventricle function variables and brain volumes and cognitive function

	Total Brain volume (cm^3)				Hippocampal volume (cm^3)			
	Model 1		Model 2		Model 1		Model 2	
	Coeff±SE	p	Coeff±SE	p	Coeff±SE	p	Coeff±SE	p
**Systolic function**								
EF (%)	-0.32±0.44	0.5	-0.41±0.46	0.4	0.002±0.003	0.4	0.0006±0.003	0.8
CI (L/ml/m^2)	22.7±7.7	**0.004**	24.3±7.9	**0.003**	0.051±0.05	0.2	0.069±0.05	0.2
TDI Sa (cm/s)	14.9±3.2	**<0.0001**	14.9±3.3	**<0.0001**	0.050±0.02	**0.01**	0.037±0.02	0.07
**Diastolic function**								
Mitral E (cm/s)	0.50±0.25	**0.05**	0.50±0.3	**0.05**	-0.002±0.002	0.2	-0.001±0.002	0.5
E:Ea	-0.80±1.4	0.6	-0.27±1.5	0.9	-0.019±0.009	**0.03**	-0.010±0.01	0.3
E:Aa	-0.40±1.9	0.8	-0.24±2.0	0.9	-0.018±0.01	0.1	-0.008±0.01	0.5
Ea:Aa	-2.27±2.0	0.9	-8.65±20.4	0.7	-0.17±0.13	0.2	-0.16±0.13	0.2
DD group	0.68±9.5	0.9	-5.14±9.6	0.6	0.026±0.06	0.7	0.024±0.06	0.7
LADI (cm/m)	-3.25±14	0.8	-0.51±15	0.97	-0.04±0.09	0.7	0.03±0.1	0.7
**Global function**								
NT-proBNP (pgml)	1.18±5.2	0.8	1.61±5.6	0.8	-0.098±0.03	**0.004**	-0.067±0.04	0.06
**Structure**								
LVMI ($g/m^{2.7}$)	0.0007±0.4	0.98	0.18±0.4	0.6	-0.002±0.002	0.4	0.0003±0.003	0.9

	CSID (z-score)				Verbal Memory (z-score)			
	Model 1		Model 2		Model 1		Model 2	
	Coeff±SE	p	Coeff±SE	p	Coeff±SE	p	Coeff±SE	p
**Systolic function**								
EF (%)	0.0015±0.002	0.5	0.0009±0.002	0.7	-0.0001±0.002	0.9	-0.0020±0.002	0.4
CI (L/ml/m^2)	-0.10±0.04	**0.005**	-0.088±0.04	**0.01**	-0.12±0.04	**0.003**	-0.11±0.04	**0.004**
TDI Sa (cm/s)	0.003±0.01	0.8	0.009±0.01	0.6	0.023±0.02	0.1	0.022±0.02	0.1
**Diastolic function**								
Mitral E (cm/s)	0.0008±0.001	0.5	-0.0016±0.001	0.2	-0.0033±0.001	**0.008**	-0.0022±0.001	0.08
E:Ea	-0.003±0.006	0.6	0.0014±0.007	0.8	-0.020±0.007	**0.003**	-0.020±0.007	**0.007**
E:Aa	0.0086±0.009	0.3	0.020±0.01	**0.03**	-0.022±0.009	**0.03**	-0.011±0.0.01	0.3
Ea:Aa	0.13±0.1	0.2	0.20±0.1	**0.03**	0.043±0.1	0.7	0.061±0.1	0.5
DD group	-0.013±0.45	0.8	-0.04±0.04	0.3	-0.019±0.05	0.7	-0.035±0.414	0.4
LADI (cm/m)	-0.023±0.07	0.7	-0.01±0.07	0.9	-0.09±0.07	0.2	-0.03±0.07	0.7
**Global function**								
NT-proBNP (pgml)	-0.0068±0.02	0.8	-0.0041±0.02	0.9	-0.045±0.03	0.07	-0.020±0.03	0.4
**Structure**								
LVMI (g/m$^{2.7}$)	0.0008±0.002	**0.01**	-0.0026±0.002	0.1	-0.0066±0.002	**<0.0001**	-0.005±0.002	**0.006**

	Visual Memory (z-score)				Working Memory (z-score)			
	Model 1		Model 2		Model 1		Model 2	
	Coeff±SE	p	Coeff±SE	p	Coeff±SE	p	Coeff±SE	p
**Systolic function**								
EF (%)	-0.0008±0.003	0.8	-0.0004±0.003	0.9	0.0032±0.002	0.2	0.0025±0.002	0.2
CI (L/ml/m^2)	0.075±0.05	0.1	0.077±0.05	0.1	-0.044±0.04	0.3	-0.027±0.04	0.5
TDI Sa (cm/s)	-0.0052±0.02	0.8	-0.0047±0.02	0.8	0.003±0.02	0.9	0.0009±0.02	0.96
**Diastolic function**								
Mitral E (cm/s)	0.0002±0.002	0.9	0.00057±0.002	0.7	0.0022±0.001	0.1	0.0027±0.001	**0.05**
E:Ea	-0.0013±0.09	0.9	-0.0005±0.01	0.96	0.0047±0.007	0.5	0.0080±0.008	0.3
E:Aa	0.010±0.01	0.4	0.016±0.01	0.2	0.0085±0.01	0.4	0.020±0.01	0.06
Ea:Aa	0.13±0.13	0.3	0.16±0.13	0.2	0.043±0.1	0.7	0.11±0.1	0.3
DD group	-0.98±0.06	0.1	-0.10±0.06	0.1	0.065±0.05	0.2	0.029±0.05	0.5
LADI (cm/m)	0.09±0.09	0.3	0.13±0.10	0.2	-0.21±0.07	**0.004**	-0.19±0.08	**0.01**
**Global function**								
NT-proBNP (pgml)	-0.076±0.03	**0.02**	-0.083±0.04	**0.02**	-0.033±0.03	0.2	-0.026±0.03	0.4
**Structure**								
LVMI (g/$m^{2.7}$)	0.0007±0.002	0.8	0.0019±0.003	0.5	-0.007±0.002	**<0.0001**	-0.0055±0.002	**0.007**

	Fluency (z-score)				Processing Speed (z-score)			
	Model 1		Model 2		Model 1		Model 2	
	Coeff±SE	p	Coeff±SE	p	Coeff±SE	p	Coeff±SE	p
**Systolic function**								
EF (%)	0.0049±0.003	**0.05**	0.0037±0.003	0.2	-0.0038±0.002	**0.05**	-0.0036±0.002	0.06
CI (L/ml/m^2)	-0.019±0.04	0.7	-0.023±0.05	0.6	0.052±0.03	0.1	0.039±0.03	0.3
TDI Sa (cm/s)	0.031±0.02	0.08	0.030±0.02	0.07	0.036±0.01	0.8	0.0015±0.01	0.9
**Diastolic function**								
Mitral E (cm/s)	-0.0013±0.001	0.4	-0.0002±0.001	0.9	-0.00027±0.001	0.8	-0.001±0.001	0.2
E:Ea	-0.009±0.008	0.2	-0.006±0.008	0.5	0.0057±0.006	0.3	0.001±0.006	0.9
E:Aa	-0.086±0.01	0.4	-0.037±0.01	0.8	0.0006±0.008	0.9	-0.0086±0.009	0.3
Ea:Aa	-0.018±0.11	0.9	-0.082±0.12	0.8	-0.11±0.08	0.2	-0.16±0.09	0.06
DD group	0.038±0.05	0.5	0.022±0.11	0.8	-0.030±0.04	0.5	-0.012±0.04	0.8
LADI (cm/m)	-0.18±0.08	**0.02**	-0.14±0.08	0.08	0.08±0.06	0.2	0.03±0.06	0.7
**Global function**								
NT-proBNP (pgml)	-0.043±0.03	0.1	-0.036±0.03	0.2	0.063±0.02	**0.006**	0.049±0.02	**0.04**
**Structure**								
LVMI (g/$m^{2.7}$)	-0.0058±0.002	**0.004**	-0.0047±0.002	**0.04**	0.004±0.002	**0.02**	0.002±0.002	0.3

Data are β± SE: Model 1: adjusted for age, sex and ethnicity. Model 2: additionally adjusted for diabetes, hypertension, previous stroke, coronary artery disease, waist-to-hip ratio, years of education and smoking. EF: ejection fraction; CI: cardiac index; Mitral E: Mitral inflow early wave, A: mitral inflow late wave. Sa: tissue Doppler imaging peak systolic wave; Ea: tissue Doppler imaging peak early diastolic wave, Aa: tissue Doppler imaging peak late diastolic wave. LADI: left atrium diameter indexed to height; NT-proBNP: N terminal prohormone brain natriuretic peptide; LVMI: left ventricle mass indexed to height$^{2.7}$.
